# Development of a Nanostructured Lipid Carrier (NLC) by a Low-Energy Method, Comparison of Release Kinetics and Molecular Dynamics Simulation

**DOI:** 10.3390/pharmaceutics13040531

**Published:** 2021-04-10

**Authors:** Andrea C. Ortiz, Osvaldo Yañez, Edison Salas-Huenuleo, Javier O. Morales

**Affiliations:** 1 Department of Pharmaceutical Science and Technology, School of Chemical and Pharmaceutical Sciences, Universidad de Chile, Santiago 8380494, Chile; andrea.ortiz@ug.uchile.cl (A.C.O.); osvyanezosses@gmail.com (O.Y.); 2 Advanced Center for Chronic Diseases (ACCDiS), Santiago 8380492, Chile; 3 Center of New Drugs for Hypertension (CENDHY), Santiago 8380494, Chile; 4 Advanced Integrated Technologies (AINTECH), Santiago 7821207, Chile; edison.salash@gmail.com

**Keywords:** lipid nanoparticle, NLC, low-energy method, Gelucire^®^ 44/14, drug release, Franz’s cells, molecular dynamics simulations

## Abstract

Lipid nanocarriers have a great potential for improving the physicochemical characteristics and behavior of poorly water-soluble drugs, such as aqueous dispersibility and oral bioavailability. This investigation presents a novel nanostructured lipid carrier (NLC) based on a mixture of solid lipid glycerides, fatty acid esters of PEG 1500 (Gelucire^®^ 44/14), and an oil mix composed of capric and caprylic triglycerides (Miglyol^®^ 812). These NLCs were developed by a simple low-energy method based on melt emulsification to yield highly encapsulating and narrowly distributed nanoparticles (~100 nm, PdI = 0.1, and zeta potential = ~−10 mV). Rhodamine 123 was selected as a poorly water-soluble drug model and owing to its spectroscopic properties. The novel NLCs were characterized by dynamic light scattering (DLS), zeta potential, nanoparticle tracking analysis (NTA), transmission electron microscopy (TEM), differential scanning calorimetry (DSC), and colloidal stability. The drug release was determined through a dialysis bag and vertical Franzs’ cells to provide insights about the methods’ suitability, revealing similar performance regardless of their different fluid dynamics. Rhodamine 123 followed a characteristic biphasic release profile owing to the swelling of the hydrophilic polymer coating and diffusion process from the lipid core as revealed by the Korsmeyers–Peppas kinetic modeling. Moreover, to elucidate the formation and incorporation of Rhodamine 123 into the NLC core, several molecular dynamics simulations were conducted. The temperature was shown to be an important condition to improve the formation of the nanoparticles. In addition, the liquid lipid incorporation to the formulation forms nanoparticles with imperfect centers, in contrast to nanoparticles without it. Moreover, Miglyol^®^ 812 improves hydrophobic molecule solubility. These results suggest the potential of novel NLC as a drug delivery system for poorly water-soluble drugs.

## 1. Introduction

Nanotechnology has contributed to different technology fields, such as biomedical, food industry, chemistry, and other fields [1,2,3]. In pharmaceutical sciences, nanotechnology promotes drug delivery carrier development that improves the final formulations’ properties. Nanocarrier development can enhance the efficacy of active molecules that present low aqueous solubility and low tissue permeability, thereby improving their bioavailability and stability [4]. Furthermore, lipid nanoparticles are an exceptional type of nanosystem due to their affinity with poorly water-soluble drugs. Nanostructured Lipid Carriers (NLC) are a new generation of nanoparticles developed to overcome some limitations of previous lipid nanoparticles [5]. The characteristics they give in pharmaceutical formulations to active molecules include enhanced solubility, increased storage stability, improved bioavailability and permeability, controlled release, and prolonged half-life, among others [6]. These advantages are related to the NLC components’ ratio because it comprises solid lipids, liquid lipids, and surfactants suspended in aqueous media. In the literature, various materials and three types of NLC have been described [7]. 

Several techniques to produce NLC have been reported. They are divided into two groups classified by high and low energy processes [8]. For high-energy methods, ultrasonication or high-pressure homogenization are required. Therefore, manufacturing NLC with high energy methods, specific high-cost equipment is needed. On the other hand, low-energy approaches involve emulsification or evaporation methods. They comprise of more affordable techniques but consist of extraction steps where organic solvents are used to solubilize the lipid phase and the active component. This may leave solvent traces in the final formulation. 

Once the active molecule is incorporated into the nanosystem, the NLC behavior is fundamental to understanding the release process. To evaluate the NLC release performance, diverse methodologies are described. Dialysis is the most used method, consisting of a physical separation by size using a membrane with a specific pore size. The active molecule should be able to trespass the membrane when the nanosystem releases it. Nevertheless, an immobile water layer formation in the membrane that limits the release rate is usual. Hence, testing of the slow-release formulations is suggested since the membrane transport should not be the limiting effect [9]. A method adaptation is an automatic process in Franz’s cells in which a small sample amount is needed. This method is conventionally used to evaluate the in vitro release and ex vivo skin permeation of formulations. Nevertheless, Franz diffusion cells have some limitations, such as low solution hydrodynamics, poor mixing, and temperature difference between donor and receptor compartments [10]. Another critical parameter is the membrane type and MWCO because it could impact the results, since permeation kinetics may be related to the dialysis membrane porosity or the interactions between the drug molecules and membrane materials [11]. Despite the existence of previous investigations reported, a comparison between these methods at the in vitro release level from NLC has not been studied so far. 

In this work, we structured a novel NLC system by a low-energy process without using organic solvents. The nanosystem was physicochemically characterized for size and particle properties (dynamic light scattering, nanoparticle tracking analysis, and transmission electron microscopy). To determine the physical state of the components in the nanoparticle a calorimetry study was carried out. Also, to understand the nanosystem behavior for a future pharmaceutical formulation, reconstitution, colloidal, and pH stability studies were performed. On the other hand, rhodamine 123 incorporation and release studies were evaluated using two methodologies, adjusting the kinetic models. Moreover, classical molecular dynamics simulations were carried out in order to visualize and understand how every atom in the molecular system will move over time, based on a general model of physics governing interatomic interactions [12]. A homogeneous and stable formulation was obtained. The release process was governed by Fickian diffusion, and both techniques (dialysis bag and Franz’s cells) had similar release performance from NLC. Finally, the molecular dynamics simulations were used to capture a wide variety of important biomolecular processes, including the NLC self-assembly processes.

## 2. Materials and Methods

### 2.1. Materials

Gelucire^®^ 44/14 was donated (Gattefosse, Lyon, France). Miglyol^®^ 812, Tween^®^ 80, Rhodamine 123 (≥85%) were purchased (Sigma-Aldrich, St. Louis, MO, USA). Amicon Ultra^®^ 10 KDa was purchased (Merck, Darmstadt, Germany). SnakeSkin^TM^ Dialysis Tubing of cellulose membrane with a MWCO 10 kDa was purchased (Thermo Fisher Scientific, Rockford, IL, USA). A Milli Q water Ultrapure Simplicity^®^ equipment was used to produce ultrapure water used in all experiments (Merck, Darmstadt, Germany).

### 2.2. Fabrication of Nanostructured Lipid Carrier (NLC) and Loading of Rhodamine 123

A manufacturing process was adapted from a previous report [13]. The compositions and raw material used for NLC development are shown in Table 1. This method was manufactured by melt emulsification combined with an injection technique without the requirement of high energy. Briefly, the lipid phase was composed of Gelucire^®^ 44/14, Miglyol^®^ 812, and Tween^®^ 80. They were added to a round bottom flask, and the aqueous phase was prepared in another glass container, both heated at 85 °C. The aqueous phase was added to the lipid phase by slow dripping under stirring for 20 min. Finally, the formulation was cooled rapidly in a fridge at 4 °C for 30 min without stirring. To obtain NLC labeled (NLC-Rho), 0.5 mg of Rhodamine 123 was incorporated into the lipid phase, and the process continues as described above.

### 2.3. Incorporation Efficiency and Drug Loading

To quantify Rho in NLC, the formulation was ultracentrifuged in 10 kDa MWCO Amicon Ultra^®^ for 20 min at 7200 rcf. Then, free Rho was separated and quantified through spectrophotometry at 512 nm. The incorporation efficiency and drug loading percentages were determined according to the following Equations (1) and (2), respectively:

(1) $$\%\;Incorporation\;efficiency=\;\frac{Rho_{total}-Rho_{free}}{Rho_{total}}\;\times \;100\;$$

(2) $$\%\;Drug\;Loading=\frac{Rho_{total}-Rho_{free}}{Solid\;lipid+\left(Rho_{total}-Rho_{free}\right)}\;\times \;100$$

where *Rho_total_* and *Rho_free_* are initial Rho amounts, incorporated into the NLC-Rho preparation. The unbound Rho amount corresponds to the evaluated by spectrophotometry.

### 2.4. Particle Size, Polydispersity Index, Zeta Potential Analysis, and NLC Particle Concentration

Hydrodynamic diameter and polydispersity index (PdI) were determined by dynamic light scattering (DLS). The zeta potential was measured by Doppler laser microelectrophoresis. Each sample was measured three times, each measurement corresponds to the average of 11 determinations in aqueous medium (viscosity = 0.08872 cp, medium refractive index = 1.330, and sample refractive index = 1.333) with a wavelength of 633 nm with a detection angle of 173° and equilibration time of 120 s. in a Malvern Zetasizer Nano ZS equipment (Malvern Panalytical Ltd., Malvern, UK). To compare the obtained size and to determine the NCL particle concentration, Nanoparticle Tracking Analysis (NTA) was performed using a NanoSight NS300 (Malvern Panalytical, Malvern, UK) equipment. For the measurements, all samples were diluted 1:10,000 with HPLC water. The diluted samples were injected with sterile syringes into the sample chamber equipped with a 532 nm diode laser (green). All samples were measured in a single shutter and gain mode for 90 s with manual shutter, gain, brightness, and threshold adjustments at room temperature. Three measurements of each newly injected sample were performed. To determine the D10, D50, and D90 as diameters size distributions, the mean size and SD values obtained by the NTA software were based on the arithmetic values calculated with all particles analyzed. 

### 2.5. Morphology of NLC

Transmission Electron Microscopy (TEM) was performed to visualize the NLC morphology. Briefly, NLC was diluted 100 times with water, deposited on film-coated copper grids, stained with 1% phosphotungstic acid for 2 min, washed with water, and dried for 12 h at room temperature. The samples were evaluated on an Inspect F50 scanning transmission electron microscope (FEI, Hillsboro, OR, USA).

### 2.6. Calorimetric Evaluation of NLC

To determine the physical state of components in NLC, Differential Scanning Calorimetry (DSC) was performed in a DSC131 (SETARAM Inc., Cranbury, NJ, USA) equipment. NLC was freeze-dried with trehalose 5% before the DSC measurement. Each sample (1–5 mg) was placed in an aluminum pan and evaluated with a temperature gradient of 25 °C to 65 °C at 5 °C/min.

### 2.7. Freeze-Drying and Reconstitution Studies 

The NLC was diluted with cryoprotectant solution (1:1) before freezing at −80 °C overnight. Samples were lyophilized for 48 h at maximum vacuum. The dry products were solubilized with 1 mL of water and sonicated for 2 min. The hydrodynamic diameter, PdI, and zeta potential were determined for all reconstituted samples as mentioned before (Table 2).

### 2.8. Colloidal Stability

The stability of the formulations (NLC and NLC-Rho) was evaluated while being protected from light for 3 months at room temperature and 4 °C. Also, they were assessed at 45 °C for 1 month. The data for 12 weeks at 45 °C was not considered because it corresponds to an accelerated stability study. For each sample, hydrodynamic size, PdI, and zeta potential were recorded at different times. 

### 2.9. pH Stability of NLC-Rho

To study the pH stability of NLC-Rho, hydrodynamic diameter, PdI, and zeta potential were analyzed at room temperature in a range of pH conditions. Briefly, NLC-Rho was diluted in water (1:10), and the sample was titrated with HCl and NaOH 0.1 M in Zetasizer Malvern Nano ZS (Malvern Panalytical Ltd., Malvern, UK) at 25 °C.

### 2.10. Release Studies

The Rhodamine 123 (Rho) release from NLC was performed through two methodologies. First, the experiment was performed using SnakeSkin^TM^ dialysis bag (MWCO 10 KDa) in 10 mL of PBS (pH 7.4) and 400 µL of sample. Second, the method was realized in vertical Franz diffusion cells (Phoenix^TM^ DB-6) with the same SnakeSkin^TM^ membranes, 10 mL of PBS (pH 7.4), and 1 mL of sample. Both methodologies were performed at 25 °C and 37 °C under continuous stirring at 400 rpm, maintaining sink conditions throughout the whole experiment. The samples were drawn at selected time intervals for 72 h and replaced with the same volume of fresh medium in the receptor chamber. The amount of Rho was measured in triplicate by fluorescence at 525 nm excitation peak. In addition, five kinetic models (zero-order, first-order, Higuchi, and Korsmeyer–Peppas functions) were used to fit the experimental data obtained from drug release studies. The coefficient of determination (R^2^) was used to select the model with better fitting for the experimental result and the similarity factor (ƒ_2_), to compare the profile of both methodologies. 

### 2.11. Molecular Dynamics Simulations 

Molecular dynamics (MD) simulations to the four ingredients of NLC in the presence of aqueous media were performed, namely Gelucire^®^ 44/14, Miglyol^®^ 812, Tween^®^ 80, and Rho. The full geometry optimizations of all molecules were carried out with the density functional theory method by a hybrid functional B3LYP functional (Becke’s Three Parameter Hybrid Functional Using the LYP Correlation Functional) along with the 6-31+G(d) basis set [14,15]. All molecules were parameterized using the LigParGen web server and implementing the OPLS-AA/1.14*CM1A(-LBCC) force field parameters for organic ligands [16,17,18]. The simulations were carried out using an explicit solvent with the TIP3P-FB water model (≈50.000 water molecules), within the OpenMM software [19,20,21]. Starting configurations were generated in cubic boxes with lateral dimensions of 120 Å set up with three-dimensional periodic boundary conditions. The system was initially prepared by placing all the molecules at random in the simulation box using a packing molecule in defined regions of space called Packmol [22]. First, each system was minimized (40,000 steps) and equilibrated (5 ns). Then, 200-ns-long production MD simulations were performed on each system. During the MD simulations, the equations of motion were integrated with a 2-fs time step in the NPT ensemble at a pressure of 1 atm. The SHAKE algorithm was applied for all hydrogen atoms, and the Van der Waals cutoff was set to 12 Å. The temperature was maintained at 300 K for one system, and for the other three systems, it was 358 K (experimental temperature during NLC synthesis) by employing the Langevin thermostat method with a relaxation time of 1 ps. The Monte Carlo barostat was used to control the pressure of 1 atm. Long-range electrostatic interactions were considered by means of the Particle Mesh Ewald (PME) approach. Data were collected every 1 ps during the MD runs. Molecular visualization of the systems and MD trajectory analysis were carried out with the VMD software package [23].

### 2.12. Non-Covalent Interaction Index

To reveal the possible non-covalent Rho molecules in the presence of the mixed NLC interactions, such as hydrogen bonds, steric repulsion, and Van der Waals interactions, the non-covalent interaction index (NCI) was used [24,25]. The NCI is based on the electron density (ρ), its derivatives, and the reduced density gradient (s). In this work, the promolecular densities (ρpro), computed as the sum of all atomic contributions were used. The NCI was calculated using the NCIPLOT program [24].

### 2.13. Data Analysis and Statistics 

All experiments were performed in triplicate, and all the data were expressed as the mean value ± standard deviation (SD). The statistical data analysis was conducted by Student’s t-test, with a *p*-value < 0.05, using the GraphPad Prism software version 6.01. To evaluate the kinetic fit and the similarity factor (ƒ_2_), the DDSolver add-In (Microsoft Excel) program was used.

## 3. Results and Discussion

### 3.1. Low-Energy Synthesis and Physicochemical Properties of Novel NLC

**Figure 1:**
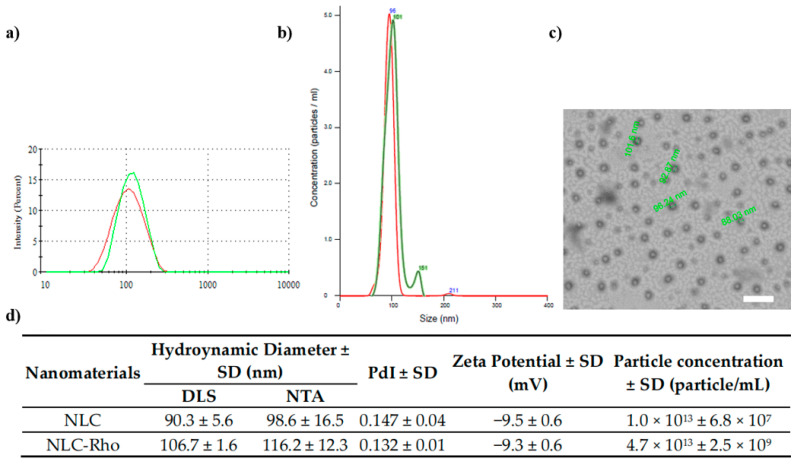
Characterization of NLC (red line) and NLC-Rho (green line). (**a**) Hydrodynamic diameter distribution, (**b**) Nanoparticle Tracking Analysis, (**c**) TEM image of NLC (the bar represents 500 nm), and (**d**) Hydrodynamic diameter (nm), PdI, and zeta potential (mV) by DLS.

Different methods have been reported to produce NLC. The most recent studies use high-energy techniques, such as ultrasonication or high-pressure homogenization [26]. The low-energy methods are based on the spontaneous formation of droplets when the ratio composition (surfactant/oil/water) is modified [27]. These methods include microemulsification, double emulsification, phase inversion temperature, coacervation, and membrane contactor [28]. Generally, the NLC synthesis by low-energy methods is usually combined with an organic solvent or stirring process [29]. The method used in this work is based on a previous report that utilizes the same solid lipid. The lipid nanoparticles obtained had a comparable size and dispersity to those obtained here; however, our manufacturing process does not use high energy such as ultrasonic cell disrupt to obtain a comparable nanoparticle size [13]. Carbone et al. synthesized a NLC loaded with ferulic acid through phase inversion temperature. Their results showed a hydrodynamic diameter and PdI values of <50 nm and ~0.3, respectively [30]. Along the same lines, Sun et al. developed a similar method to manufacture NLC, with hydrodynamic size results of <100 nm and PdI >0.3 [31]. In comparison, our NLC obtention strategy needs low energy but does not use organic solvents and it is a quick process compared with other reported methodologies. The obtained nanoparticles have ~100 nm with PdI lower than 0.15 (Figure 1). 

In pharmaceutical applications, the nanoparticle size and dispersion are crucial parameters to assure an optimal formulation behavior in terms of stability, arrival at the site of action, delivery, and safety. In the high-energy method, sonication and high-pressure homogenization are key steps to decrease nanoparticle size [32]. In the low-energy methods, the temperature is a determining factor in reducing the size and dispersion. In this context, the fundamental steps in the low-energy process are (i) high and constant temperature that permits the solid lipids, surfactant, oil, and cargo an adequate mixing [33]; (ii) slow dripping, continuous agitation, and high temperature that allows the O/W drop to structure with an optimal size dispersion in the nanometric range [34]; (iii) high-temperature gradients to facilitate the rapid lipid crystallization, preventing aggregation. Moreover, at high temperatures, the hydrophilic head of the non-ionic surfactant begins to be more dehydrated than the hydrophobic tail, which is why W/O formulations are favored. However, when the temperature is lowered, the surfactant head is more hydrophilic than the tail and forms O/W formulations [35]. Therefore, the cooling process is an essential step because it allows solid lipid solidification and helps maintain the size and monodispersion [36].

Regarding the process described above, Gelucire^®^ 44/14 has a fundamental role in the formulation due to the amphiphilic self-assembly characteristics, forming a fine dispersion in contact with aqueous media [37]. The Gelucire^®^ 44/14 melting point is 44 °C; therefore, nanoparticles can be solidified without requiring a large temperature difference. Furthermore, it can be used as a binder in melt methods, and due to its thermoplastic behavior, it allows the rapid formation of stable crystalline structures [38]. Mygliol^®^ 812, a liquid lipid component at room temperature, contributes to the mixing process, allowing the components dissolution and the incorporation of higher Rho quantities in the NLC core [39]. Finally, Tween^®^ 80, contributes to the NLC formation and stabilization in aqueous media and, with Gelucire^®^ 44/14, maintains the NLC stability over time [40]. These results are consistent with the observed in the molecular dynamics simulations (Section 3.9).

In general, physical parameters such as a spherical shape, low size distribution, and a zeta potential range of up to ±35 mV are acceptable characteristics to obtain improved stability and low toxicity [41]. In this study, we used different materials and components ratios (Table S1). Accordingly, we selected a formulation based on these parameters (Figure S1). The NLC and NLC-Rho nanosystems had a hydrodynamic diameter measured by DLS, around 90 and 100 nm, respectively. The sizes were consistent with the NTA determination, and the obtained zeta potential was <−9.5 for both systems (Figure 1). As Pimentel-Moral et al. considered, the NCL and NCL-Rho nanosystems were characterized as monodisperse with a PdI of <0.15 [42]. 

A manufacturing advantage of using high-energy methods is the low size dispersion obtained. The sonication and high-energy mixing allow the obtention of PdI around 0.1–0.3 [43]. In our formulation, the low size distribution is attributed to the cooling process. The solid lipids solidify at temperatures below 44 °C, and the lipid droplets formed in the process partially solidify and prevent the coalescence [44]. The negative zeta potential was attributed, in part, to the surfactant and some free fatty acids in the interface, such as derived from the glyceride hydrolysis present in Gelucire^®^ 44/14 [45]. The obtained zeta potential indicates a low nanoparticle electrostatic repulsion, thus slightly preventing the aggregation. Nonetheless, the free esters of PEG, free PEG in Gelucire^®^ 44/14 and Tween^®^ 80 contribute to forming hydrophilic nanoparticles and improve the stability of NLC by steric interaction, hydration in the surface layer, and resistance to flocculation and coalescence [46]. Despite this, the surface charge is a crucial factor for biocompatibility and can determine nanoparticle behavior in a biological context. Processes such as cell-membrane interactions, internalization, opsonization, and biological fluid stability can be governed by the charge of the nanosystem [47]. Thus, positively charged nanoparticles can internalize it more actively than neutral or negative nanoparticles [48]. However, nanoparticles with a slightly negative surface tend to accumulate in tumor tissues more efficiently [49]. On the other hand, positive nanoparticles can activate faster immune responses than neutral or negative nanoparticles [50]. 

Both NLC and NLC-Rho were kept in the nanometric range because the formulations have a D90 less than 140 nm. Besides, appropriately dispersed and spatially separated nanoparticles were visualized in the video obtained from the Brownian motion of NLC in suspension. The particle concentration was determined by NTA. Both formulations presented near 1013 particle/mL (Figure 1b). NTA determination has some advantages compared with the DLS, mainly the precise sizing, reliable size distribution, and easy detection of contaminants [51]. Also, with NTA it is possible to visualize the nanoparticle trajectory following the Brownian movement in a real-time period. This is possible while the particle is visible or intersects with an adjacent particle [52]. For these reasons, it is always proper to evaluate a nanosuspension by NTA and compare it with the DLS method. NTA is a useful tool in the pharmaceutic field related to nanotechnology because it allows knowing the particle concentration, which gives us valuable information to understand the doses of the active molecules carried by the nanosystems.

### 3.2. Morphology

To determine NLC shape and morphology they were visualized by TEM. It was observed that NLC has a spherical shape and monodisperse size distribution among the evaluated samples, indicating the reproducibility of the process (Figure 1c). The spherical shape results due to the homogenization process, because of the reduced surface tension provoked by the presence of Tween^®^ 80, and spherical nanoparticles formed are stabilized after the cooling process. The minimum interfacial energy facilitates the formation of a closer spherical bilayer. However, nanoparticle self-assembly is not spontaneous. The steric or ionic repulsion between hydrophobic and hydrophilic materials are the promoters, requiring the application of external energy [53]. A slight difference can be noted between the obtained sizes by TEM and DLS. This difference is because the TEM images reveal the size of the solid-state after a drying process. Thus, contributing to nanoparticles acquiring a flattened shape and increased size [45]. In contrast, DLS measures the nanoparticle in aqueous solutions, tending to exhibit a larger size due to the solvation layer [54].

### 3.3. Physical State Evaluation of NLC 

**Figure 2:**
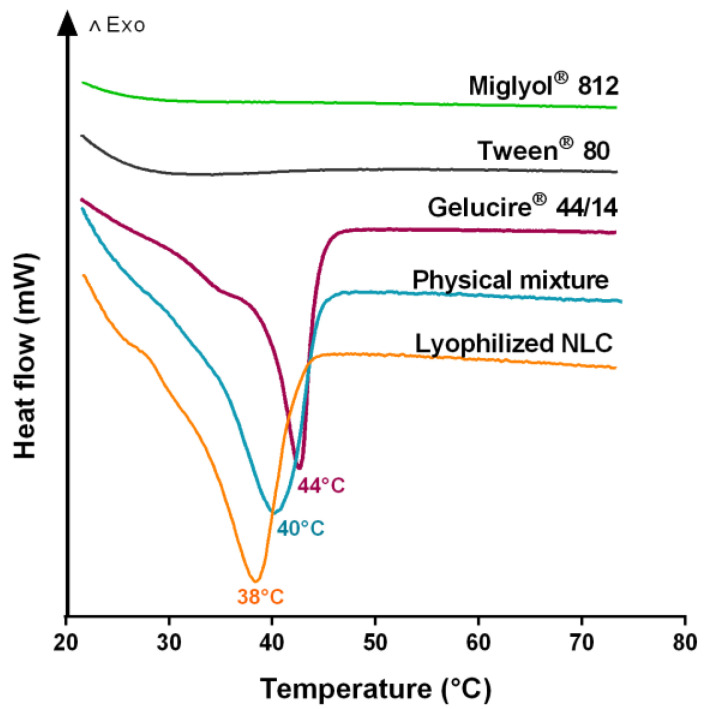
Differential scanning calorimetry (DSC) thermograms of Miglyol^®^ 812, Tween^®^ 80, Gelucire^®^ 44/14, physical mixture of NLC and lyophilized NLC. The figure was produced with a representative profile of repetitions (*n* = 1).

Differential Scanning Calorimetry is a powerful approach to study the components’ physical state in a nanoparticle formulation. The pure materials, physical mixture, and NLC thermogram profiles are shown in Figure 2. Gelucire^®^ 44/14 has the melting point at 44 °C. In the case of Gelucire^®^ 44/14 mixture, a displacement of the melting point from 44 °C to 40 °C, and 38 °C in the lyophilized NLC was observed. This suggests that the mixture between Miglyol^®^ 812 and Tween^®^ 80 could partially solubilize the solid lipids and favor an earlier fusion. Moreover, lyophilized NLC and physical mixture revealed a different behavior, observing an earlier fusion in the lyophilized system. This difference suggests a closer component interaction due to the nanoparticle structuration. During the manufacturing process, the solid lipids, liquid lipids, and surfactant are in close contact at high temperatures. Then, when the temperature drops abruptly, the components are homogenized and kept in close contact.

For this reason, as the temperature increases, the near contact between components decreases the melting point. Regarding the solubility of Gelucire^®^ 44/14 in the presence of Tween^®^ 80 and Miglyol^®^ 812, some reports have evaluated it by X-ray crystallography. In this context, Damian and coworkers have reported that Gelucire^®^ 44/14 has a transition phase at 19.23 °C and 23.38 °C [55]. In another research, Caon et al. reported that Gelucire^®^ 44/14 has a crystalline structure in mixture with Tween^®^ 80. Consequently, the surfactant does not solubilize the solid lipids in these conditions [56]. However, Čerpnjak et al., in 2014, studied a similar formulation, composed of Gelucire^®^ 44/14, Mygliol^®^ 812, Peceol^TM^, and Solutol^®^HS 15. Peceol^TM^ is an oil, composed of glycerides with long-chains, similar to one of the components of Gelucire^®^ 44/14, and Solutol^®^HS 15 has a similar HLB than Tween^®^ 80, 16 and 15, respectively. According to these approaches, the crystalline structure of Gelucire^®^ 44/14 was not visible in the formulation with Miglyol^®^ 812 and surfactants. Thus, this liquid lipid may improve the solubility of Gelucire^®^ 44/14 [57]. 

### 3.4. Freeze-Drying and Reconstitution Studies 

**Figure 3:**
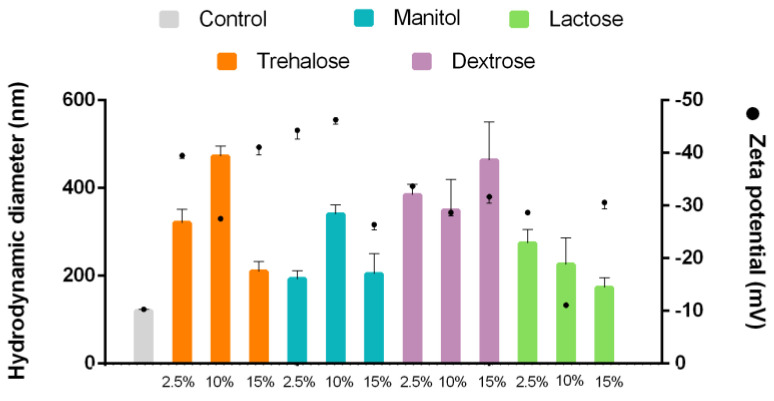
Hydrodynamic diameter and zeta potential of control and reconstitution of NLC with different concentrations of cryoprotectants. *n* = 3 ± SD.

Solid dosage forms are preferred due to their greater stability over time. As such, it is relevant to evaluate the drying and reconstitution process of the formulations. Lyophilization is one of the most common techniques to remove the aqueous medium from a suspension. It consists of sublimation in which the sample undergoes stress in the freezing and drying process. Therefore, cryoprotectants are used to preserve the nanoparticles avoiding possible agglomerations [58]. Carbohydrates have been widely used as cryoprotectants due to their ability to inhibit water crystallization. We used four types of this category, evaluating different concentrations (Table 2) [59]. In general, with a higher concentration of cryoprotectants, the system will reconstitute better [60], which was consistent with our results. Specifically, the highest concentrations of trehalose, mannitol, and lactose show similar behavior, and nanoparticles with similar characteristics to the control were obtained (Figure 3). On the contrary, all the formulations prepared with dextrose resulted in nanoparticles with high hydrodynamic diameters close to 400 nm, revealing aggregation. However, they have a zeta potential of −30 mV, which indicates an electrostatically stable system. The carbohydrate cryoprotectant role lies in the hydroxyl group’s availability. The generation of hydrogen bonding sites per molecule directly relates to depressing the freezing points at the nanosystem’s edges. Because of this, trehalose, lactose, and mannitol, with 8, 8 and 6 available hydroxyl groups, respectively, had better performance than dextrose with only 5. 

### 3.5. Incorporation Efficiency and Drug Loading

Nanoformulations play essential roles in carrying drugs with low permeability and/or solubility (BCS type II and IV) and the subsequent controlled release. In this research, Rhodamine 123 (Rho) was selected as a drug model due to its high lipophilicity, low solubility in aqueous media (BCS type II), and the opportunity to follow it by fluorescence. Usually, Rho is incorporated in nano/microparticles to label and monitor in vivo studies [61]. Rho has a high extinction coefficient, thus does not require elevated concentration to follow it and determine the delivery ratios. After NLC-Rho ultracentrifugation, unbound Rho was quantified by Uv–vis, and 418 µg/g solid lipid was determined. After the free-Rho separation, the incorporation efficiency and drug loadings were 93.5% and 0.03%, respectively. Rho was highly incorporated due to its affinity to lipids than the aqueous phase. Besides, NLC forms an imperfect core and an amorphous matrix; this also allows for a greater incorporation of lipophilic molecules because it can accommodate molecules and prevent their abrupt drug escape in storage conditions [62]. 

### 3.6. Colloidal Stability

**Figure 4:**
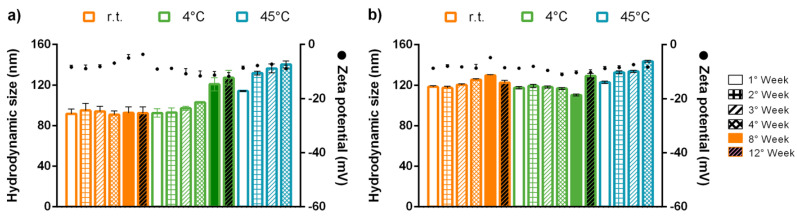
Colloidal stability studies of (**a**) NLC and (**b**) NLC-Rho at room temperature and 4 °C for 12 weeks and 45 °C for 4 weeks. Parameters evaluated were hydrodynamic size (nm), and zeta potential (mV). *n* = 3 ± SD.

The studies on lipid nanoparticle stability indicate a general tendency toward instability over short times. In general, authors refer to instability due to the crystal growth by Ostwald ripening and mushroom-like arrangement of the short chains of PEG [63]. In our case, we used a solid lipid with a long chain of triglycerides, and the cold process in the fabrication prevents the Oswalt ripening. On the other hand, the nanosystem’s physical stability is a critical characteristic. Based on visual examination, all formulations were still homogeneous in the long term, and no visible free lipids or system rupture was evidenced. We determined that NLC at room temperature and 4 °C for 4 weeks did not change the size (Figure 4). When NLC was incubated at 45 °C a size increase of ~40 nm was evidenced, attributed to agglomeration. As shown in Figure 2, the thermogram of lyophilized NLC has a fusion peak at 38 °C; therefore, nanoparticles begin a state of change due to the fusion process. However, PdI remained constant in a narrow range of 0.1–0.2. The size increase possibly corresponds to a slight agglomeration or due to a homogenous agglomeration growth [64]. The size increasing over time can be mediated by gravitational separation, flocculation, or Ostwald ripening mechanisms. To avoid Oswald ripening, it is necessary to incorporate a non-polar agent, with oil phase solubility but insoluble in the aqueous phase. Some of these agents can be triacylglycerols, diacylglycerols, monoacylglycerols, and free fatty acids. Gelucire^®^ 44/14 is composed of a long chain of triacylglycerides, mono- and di-fatty acid esters of PEG 1500, that inhibit the Oswald ripening process and decrease the size distribution [65]. Both formulations kept an average of −8 mV in charge by zeta potential, suggesting possible colloidal instability due to the closeness to neutrality. Nevertheless, it was evidenced that the nanosystem remains stable over time, explained due to the steric distancing between the nanoparticles produced by the formulation’s components. In general, polymeric material induces steric stabilization. In the case of lipid nanoparticles, this role is usually accomplished by surfactant. However, the main component in this formulation is Gelucire 44/14, composed of polyethyleneglycol that improves the stability along with Tween^®^ 80. Thermodynamically steric stabilization is determined by estimating Gibb’s free energy, and the strong enthalpic interaction is due to the efficient solvation between the solvent and the stabilizing agent (surfactant). In addition, a nonionic surfactant can give a solvation barrier to a close contact. Then, stabilization segments cannot interpenetrate with another nanoparticle producing a steric stabilization. The solvation barrier is considered an additional force in the DLVO model. Modern DLVO variants include calculations of Van der Waals forces, electrostatic forces, and solvation forces. Accordingly, the nanosystem is stabilized by the electrostatic effect and steric hindrance NLC and NLC-Rho are presented an interesting colloidal stability.

### 3.7. pH Stability of NLC-Rho

**Figure 5:**
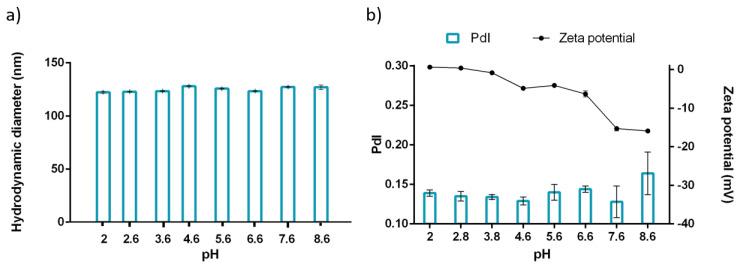
pH stability of NLC-Rho, parameters evaluated by (**a**) Hydrodynamic diameter and (**b**) PdI and zeta potential (mV). *n* = 3 ± SD.

Given the potential administration via the oral route, the NLC behavior at different pH present in the gastrointestinal tract gives us an insight into the real condition of the system applicability. The gastrointestinal tract has a pH range from acidic as 2 and slightly basic as 8, which can affect the nanosystem structure. In the evaluated pH range, the hydrodynamic diameter did not change drastically, but PdI increased until 0.2; this was attributed to a change in the superficial charge at lower pH (Figure 5a,b). Tween^®^ 80 is a nonionic surfactant and acts as a stabilizer, however, it has a negative charge in the interface, because of the differential adsorption of the hydroxyl ion (OH^−^) and hydrated oxonium ion (H_3_O^+^). The polyoxyethylene group was neutralized, then the zeta potential increased, which could allow the nanoparticle aggregation, increasing the PdI [66]. In 2017, Park et al. investigated about vitamin D3 incorporation in NLC. They observed that the NLC zeta potential at pH 2 and pH 10, changed from ~0 mV to −30 mV, respectively. The authors suggest that this is due to anionic species’ presence on the surface [67]. Ozturk et al. evaluated the influence of different carrier lipid types on the bioaccessibility of the D3 vitamin. In the research, they described that the generation and release of anionic free fatty acids from the matrix increase in an alkaline environment and contribute to a negative surface [68]. Therefore, the nanosystem surface charge variations can be used to direct the system along the gastrointestinal tract. At an acidic pH, NLC has a slightly neutral charge, which allows it to have closer contact with the gastric mucosa (negatively charged). On the other hand, at a basic pH similar to the final third of the gastrointestinal tract, NLC can be administered locally and ensure improved electrostatic stability of the nanosystem.

### 3.8. Release Studies

**Figure 6:**
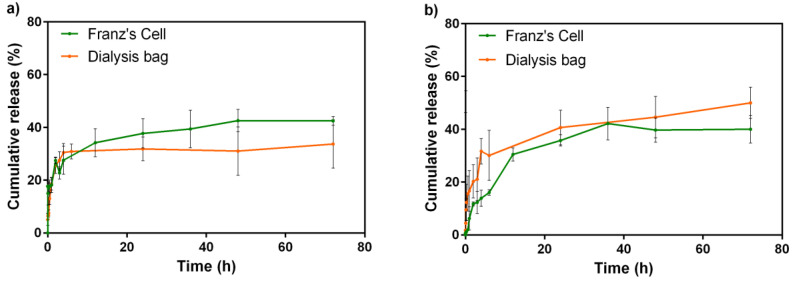
Cumulative release of Rhodamine 123 (%) at (**a**) 25 °C and (**b**) 37.5 °C during 72 h. *n* = 3 ± SD.

To evaluate the NLC-Rho release behavior, dialysis bag and vertical Franz’s cells methods were performed. Dialysis bag is a gold standard method to determine the molecule release from different kinds of nanoparticles. Franz’s cells are usually used to evaluated skin permeation, nevertheless, is an instrument that allows release studies from a matrix [9]. It is an automatic method; hence, temperature, stirring, sampling time control, and reproducibility are more precise than dialysis bags. Consequently, the variability of the results is less in Franz’s cells than dialysis bag method. Also, the dialysis membrane was selected with an adequate pore size to allow a free drug diffusion and not the nanosystem, while avoiding membrane-drug interaction. Therefore, the release profile is mainly attributed to the rhodamine 123 diffusion from the lipid matrix instead of a rhodamine 123-membrane interaction that could contribute to the observed profile. The membrane superficial area in dialysis bag and Franz’s cells was 1.38 cm^2^ and 0.63 cm^2^, respectively. Both experiments were performed at pH 7.4 for 72 h and the results show that the Rho release at 25 °C through dialysis bag and Franz’s cells were 33.7% and 42.5%, respectively (Figure 6a). In the case of 37 °C by dialysis bag and Franz’s cells were 49.9% and 40.3%, respectively (Figure 6b). 

Drug release from nanoparticles has different mechanisms depending on their composition, including matrix erosion, diffusion, or swallowing. In lipid nanoparticles, the drug release process is mainly governed by matrix diffusion and erosion [69]. Specifically, in NLC, the incorporation of a liquid lipid component in the formulation results in a less-ordered structure that prevents drug expulsion [70]. Regarding kinetic fit studies, were performed to zero order, first order, Higuchi and Korsmeyer–Peppas, resulting from this last how the better fit. In the case of the release at 25 °C, the process could be dominated by Fickian diffusion (Case I) (*n* ˂ 0.43) (Table 3) [71], this indicates that the diffusion process is faster than the matrix relaxation. About the release at 37 °C, the process is dominated by anomalous diffusion (0.43 ˂ *n* ˂ 0.85), therefore, diffusion is not the only process involved because the matrix is partially altered [72]. These results are concordant because, at 25 °C, the matrix is in the original state, Gelucire^®^ 44/14 continuous in solid-state and permit the Rho diffusion. Instead, at 37 °C, the matrix experiments physical changes due to the higher temperature, therefore Rho diffuses, but the structure becomes more labile. Furthermore, similar to our evaluation, other researchers have conducted kinetic studies of NLC. Alam et al. described a NLC that released 84% simvastatin in 24 h, and the best fit model was Korsmeyer–Peppas (R^2^ = 0.985 and *n* = 0.49) [73]. In a recent study, Eh Suk et al. evaluated several NLC formulations, concluding that even though Korsmeyer–Peppars is a model for polymeric matrices, it well represents release from NLC [74].

Additionally, we used the similarity factor (ƒ_2_) to determine whether both methodologies are interchangeable to evaluate drug release. Regarding FDA guidelines, ƒ_2_ values greater than 50 (50–100) show similarity in dissolution profiles. Empirically, this is possible if at each point the difference is less than 10% [75]. We compared and evaluated ƒ_2_ between the profiles from dialysis bag and Franz’s cells at 25 °C and 37 °C, obtaining 56.01 and 50.57, respectively. According to established parameters, both methodologies can be used to determine the drug release from NLC at 25 °C and 37 °C. It will permit a manual process to be carried out using an automatic method and obtain results in less time. However, both values are within the limit, thus, is difficult to determine if both methodologies are interchangeable reliably. These results indicate that the NLC demonstrated similar drug release in both methods and they are not suitable to show the differences in the drug release process [76]. 

Once the tests finished, hydrodynamic size, PdI and zeta potential were evaluated to determine if the nanoparticles lost their structure, agglomerated, or changed their surface charge. We observed that the hydrodynamic size increase ~20 nm, this value is attributed to a slight agglomeration (Table 4). As mentioned above and showed in DSC thermograms, the PdI values increase at 37 °C, this could be favored at high temperatures and near the melting point of the solid lipids, allowing the nanoparticles to fuse each other. On the other hand, as mentioned earlier (Section 3.6), the zeta potential was −18 mV, more negative due to the pH of evaluation (pH 7.4). In literature, it has been reported that high salt concentrations cause a reduction of the diffuse layer, then resulting in a decrease of the zeta potential to the neutrality and diminishing the electrostatic repulsion [77]. However, the surface covering of PEG avoids the possibility of perturbing the diffuse layer. Therefore, it shows an increase in PdI due to a weak NLC aggregation after the experiment. 

### 3.9. Molecular Dynamics Analysis 

In order to better understand the self-assembly behavior of the NLC studied, a molecular modeling of the mixtures was performed. The evaluated systems were named MD-1 corresponding to Miglyol^®^ 812, Tween^®^ 80, and Gelucire^®^ 44/14 at 300 K; MD-2, consisting of Tween^®^ 80 and Gelucire^®^ 44/14 at 358 K; MD-3 which is Miglyol^®^ 812 absent at a temperature of 358 K, and MD-4 corresponding to Miglyol^®^ 812, Tween^®^ 80, and Gelucire^®^ 44/14 in the presence of Rho at 358 K (Table 5). These models were selected to distinguish the effects of electrostatic and hydrophobic interactions on the molecular components self-assembly of the mixed NLC. 

**Figure 7:**
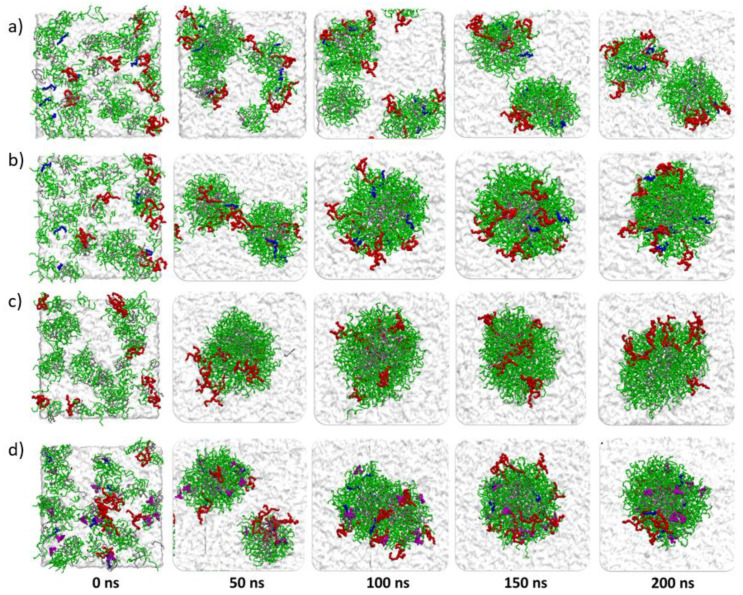
Trajectory snapshots of self-assembly process of (**a**) MD-1 (Miglyol^®^ 812, Tween^®^ 80 and Gelucire^®^ 44/14 at 300 K), (**b**) MD-2 (Miglyol^®^ 812, Tween^®^ 80 and Gelucire^®^ 44/14 at 358 K), (**c**) MD-3 (Tween^®^ 80 and Gelucire^®^ 44/14 at 358 K) and (**d**) MD-4 (Rhodamine 123, Miglyol^®^ 812, Tween^®^ 80 and Gelucire^®^ 44/14 at 358 K) in the aqueous medium at different simulation times. Color representations of Miglyol^®^ 812 (blue), Tween^®^ 80 (red), Gelucire^®^ 44/14 (green and gray) and Rhodamine 123 (purple).

The molecular simulations showed that only the MD-2, MD-3, and MD-4 systems at 358 K were stable in the self-assembly process during the 200 ns (Figure 7b–d), whereas MD-1 mixture at a temperature of 300 K was not observed during the 200 ns the self-assembly process to form only one mixed NLC (Figure 7a). 

These results are in concordance with what we observed experimentally since, at lower temperatures, the nanoparticle formation is less effective (Section 3.1). The mixed NLC formed at 358 K is not entirely spherical as they have ellipsoidal components. The molecular components of Gelucire^®^ 44/14, such as lauric acid PEG, are arranged around the hydrophobic center of the lipid nanoparticle formed by Mono-, Di-, and Tri-glyceride lauric acid. This molecular mechanism that produces hydrophobicity is one of the most important factors determining the thermodynamic stability of micelles and biological membranes. Lauric acid PEG on the surface of the NLC allows the torsional movement by which the hydrocarbon chains of lauric acid move in different directions while they associate with other chains of lauric acid PEG. The polar section of Lauric acid PEG can form hydrogen bonds with water molecules in the hydration layer. These hydration water molecules also form a weak hydrogen bond with those in the water. The terminal carbons of Lauric acid interact toward the center of the hydrophobic nucleus with Mono-, Di-, and Tri-glyceride lauric acid, differentiating the amphiphilic surface and the hydrophobic nucleus from the mixed NLC. With the amount of components molecules simulated, the average diameter of the mixed nanoparticles formed during the 200 ns was 9.2 nm. The molecules of Miglyol^®^ 812 undergo Van der Waals type interactions between the hydrocarbon chains and the polar portions of the surface of the mixed NLC constituted by lauric acid PEG, which allows a sufficiently strong anchorage to limit its exchange with the aqueous medium (Figure 7c,d). Consequently, Miglyol^®^ 812 is found in small concentrations in water, and practically all its interactions come from hydrophobic areas or near the amphiphilic lauric acid PEG interface. The configuration of Tween^®^ 80 in the mixed NLC shows its interaction on the polar PEG surface of lauric acid PEG (Figure 7b–d). This configuration experiences interactions of hydrogen bonds and Van der Waals forces between the ethylene oxides of the chains and the polar portion of the lauric acid PEG.

**Figure 8:**
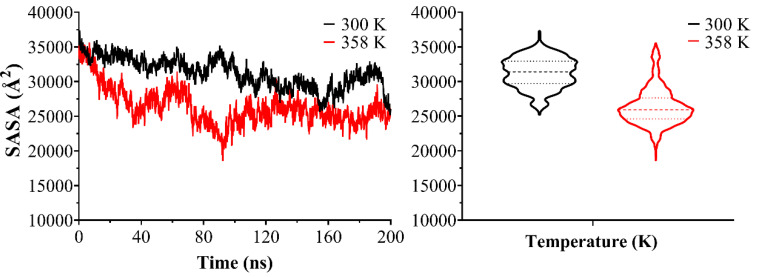
Solvent accessible surface area (SASA) as a function of simulation time for Mono-, Di- and Tri-glyceride Lauric acid at 300 K (black line) and 358 K (red line) during the self-association process.

The aggregation of hydrophobic molecules in the aqueous media is known to be driven by unfavorable contact between water and their hydrophobic domains [78]. It has been shown that as the self-association proceeds, the Solvent Accessible Surface Area (SASA) of the hydrophobic solute is constantly limited in a linear correlation with the unfavorable contacts [79,80,81]. Thereby, SASA can be used to semi-quantitatively track the self-aggregation of non-soluble agents in the aqueous solvent and see the temperature effect on this self-assembly process. To quantify the self-assembly process, the hydrophobic core of Gelucire^®^ 44/14 was considered, and the SASA profiles were measured as a function of the simulation time. The SASA profiles of the self-assembly of Mono-, Di-, and Tri-glyceride are shown in Figure 8. Lauric acid at a temperature of 300 K and 358 K, showing for the mixed NLC system at a temperature of 300 K greater accessibility to the solvent than the system at a temperature of 358 K, indicating greater self-aggregation. This is confirmed by the progressive decrease in the average number of water particles around each hydrophobic core molecule as SASA becomes more limited. The pattern of SASA variations indicates the staggered formation of larger aggregates from smaller ones, resulting in the system at a temperature of 358 K, a complete mixed NLC system, as also observed in the trajectories.

**Figure 9:**
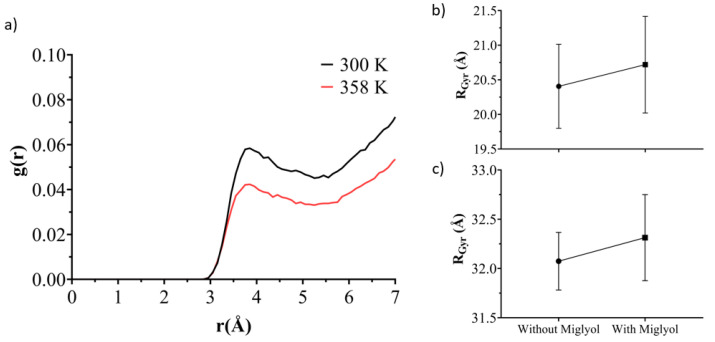
(**a**) Average radial pair distribution functions (RDF) for oxygen from the water against Lauric acid PEG tail at 300 K (black line) and 358 K (red line). The radius of gyration of (**b**) Mono-, Di- and Tri-glyceride Lauric acid (hydrophobic core) and (**c**) Lauric acid PEG (amphiphilic surface), in the presence and absence of Miglyol^®^ 812 at 358 K.

The radial density functions for the tail of lauric acid PEG and oxygen of water molecules from the mixed NLC at a temperature of 300 K and 358 K show in Figure 9a greater interaction between lauric acid PEG tails with water molecules when the temperature is 300 K; this is due to the poor self-aggregation process to form a complete mixed NLC. These results are complemented by those obtained in the SASA profiles. The obtained results from the gyration radius (RGyr) give us an insight about how the parameter that gives information on the lipid nanoparticle mass distribution around the center of gravity is an important parameter and can be influenced by Miglyol^®^ 812, especially when NLC can adopt several conformations (Figure 9b,c).

**Figure 10:**
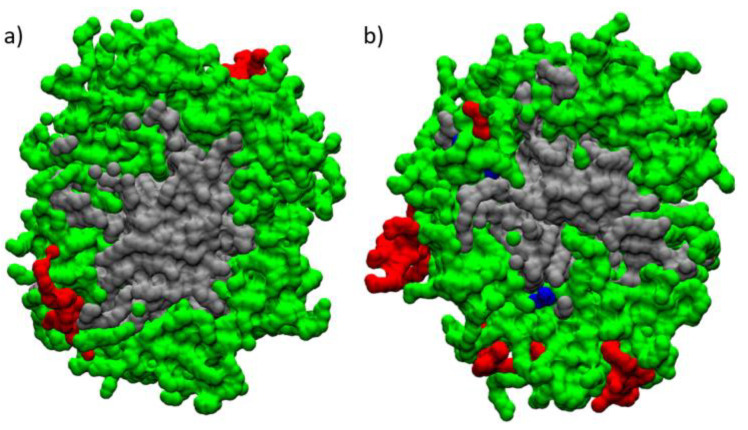
Snapshot of the mixed micelles of Tween^®^ 80 (red surf) and Gelucire^®^ 44/14 (green and gray surf) in the (**a**) absence and (**b**) presence of Miglyol^®^ 812 (blue surf) at 358 K, along with a cross-sectional view 200 ns into the simulation trajectory.

As mentioned above, a mixture of solid and liquid lipids allows a nanoparticle formation with an imperfect core. According to the obtained results, the Miglyol^®^ 812 presence in the nanoparticle structure forms a less compact core, therefore, more imperfect (Figure 10). The mixed NLC system without Miglyol^®^ 812 tends to compact and self-aggregate faster than the system with Miglyol^®^ 812. This result is consistent with what is shown in Figure 7b,c, in which the mixed NLC system without Miglyol^®^ 812 at 45 ns completely self-assembles into a single system. Although the NLC formation with Miglyol^®^ 812 is slower, incorporating this material has several advantages. Miglyol^®^ 812 improves hydrophobic molecules solubility, increases the incorporation efficiency, and different proportions of solid/liquid lipids allow the molecules release modulation from the matrix. In order to consider the reported values of (RGyr) as significant, a Student’s t-test with a *p*-value <0.05 was carried out.

**Figure 11:**
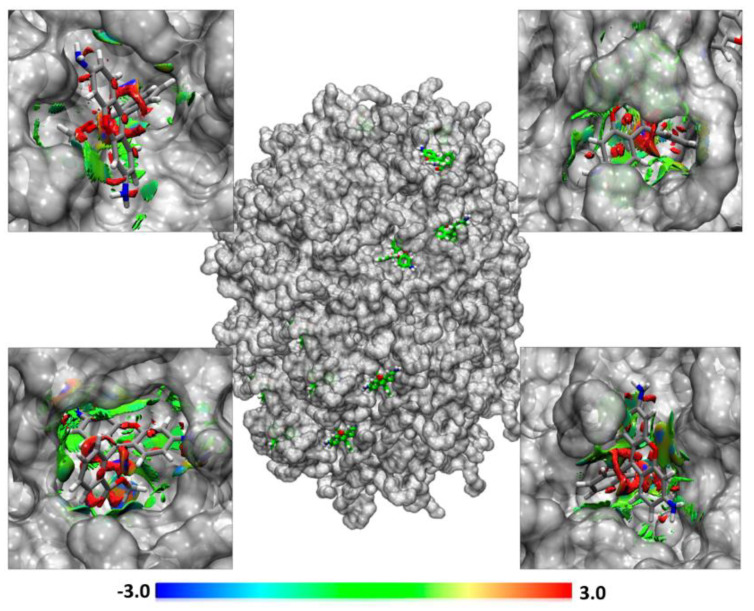
NCIPLOT isosurface gradient (0.6 au) between Rhodamine 123 and mixed NLC of Miglyol^®^ 812, Tween^®^ 80 and Gelucire^®^ 44/14. The surfaces were colored on a blue-green-red scale according to the strength and type of interaction. Blue indicates strong attractive interactions, green indicates weak Van der Waals interactions, and red indicates a strong non-bonded overlap.

For the mixed system MD-4, the molecular simulations showed high stability in the self-assembly process, and in the Rho incorporation during the 200 ns (Figure 7d) at a temperature of 358 K. Rho 123 molecules interact with the polar phase of lauric acid PEG of Gelucire^®^ 44/14. For the system with Rho molecules, NCIPLOT isosurface graphs revealed that hydrogen bonds are accompanied by weak and hydrophobic interactions like Van der Waals (Figure 11). The hydrogen bond interaction between Rho and the proposed NLC system is promoted by the lauric acid PEG esters with the two ammonium groups of Rho. Weak interactions are made between the Rho rings and the methyl groups of the lauric acid PEG molecule. These interactions give good stability of Rho 123 on the polar surface of the NLC system.

## 4. Conclusions

In the current research, a nanostructured lipid carrier was successfully manufactured by a low-energy method and characterized by different methodologies. In the formulations studied, a low size distribution was obtained which was maintained in stability studies. Furthermore, the hydrodynamic diameter of the formulation was stable at different pH, therefore, the NLC obtained can be an attractive candidate for oral pharmaceutical formulations. Besides, a poorly-water soluble model molecule was effectively incorporated. The drug release was studied using two different methodologies, these being possibly replaceable under the conditions studied. In all the conditions evaluated, the NLC-Rho release was ~40% and the Korsmeyer–Peppas fit as the best mathematical model. However, at different temperatures, the release is dominated by different mechanisms due to physical nanoparticle changes. On the other hand, dynamic molecular simulation studies have shown that the NLC formation at high temperatures improves the component compaction and the incorporation of oil in a lipid formulation creates an imperfect core and, therefore, improves the drug loading capacity. Finally, this nanosystem could be an interesting candidate to deliver molecules of low aqueous solubility through the oral route section.

## Figures and Tables

**Table 1:** Raw materials to fabricate the selected nanostructured lipid carrier (NLC) formulation.

Raw Materials	% p/p Components	Role
Solid lipid (Gelucire^®^ 44/14) Liquid lipid (Miglyol^®^ 812)	4 1	Lipid component
Tween^®^ 80	2	Surfactant
Water	93	Vehicle

**Table 2:** Cryoprotectants used in the freeze-drying.

Cryoprotectant	Concentration (%)
Trehalose	2.5, 10, 15
Mannitol	2.5, 10, 15
Dextrose	2.5, 10, 15
Lactose	2.5, 10, 15

**Table 3:** Kinetic fit evaluation to NLC-Rho at 25 °C and 37 °C in dialysis bag and vertical Franz’s cell. In equations Q_0_ and Q_t_ is the initial amount of Rho and the amount of Rho dissolved at time t, respectively. Q_t_/Q_∞_ is the fractional release of Rho, K_0_ is the zero order constant, K_1_ is the first order constant, K_H_ is the Higuchi constant, k_KP_ is the Korsmeyer-Peppas constant and n is the diffusional exponent.

Kinetic Models		Dialysis Bag		Franz’s Cell	
		25 °C	37 °C	25 °C	37 °C
Zero order $Q_{t}=Q_{0}+K_{0}t$	K	1.92	2.20	2.48	1.83
	R^2^	−1.16	0.78	−1.93	0.77
First order $\ln Q_{t}=\ln Q_{0}+\;K_{1}t$	K	0.03	0.03	0.03	0.02
	R^2^	−0.45	−0.05	−1.50	0.86
Higuchi $Q_{t}=K_{H}\surd t$	K	10.08	10.75	10.43	7.43
	R^2^	0.46	0.73	−0.19	0.96
Korsmeyer–Peppas $\frac{Q_{t}}{Q_{\infty }}=\;K_{k}t^{n}$	n	0.25	0.59	0.15	0.56
	k_KP_	17.53	16.83	22.08	6.43
	R^2^	0.87	0.94	0.93	0.97

**Table 4:** Evaluation of NLC-Rho after release studies (72 h). Parameters evaluated were hydrodynamic size (nm), PdI and zeta potential (mV). *n* = 3 ± SD.

Methodology		Hydrodynamic Size (nm ± SD)	PdI	Zeta Potential (mV ± SD)
NLC-Rho	*t* = 0	106.7 ± 1.6	0.1 ± 0.01	−9.3 ± 0.6
Dialysis bag	25 °C	118.5 ± 1.7	0.1 ± 0.01	−18.4 ± 1.6
	37 °C	115.1 ± 2.8	0.3 ± 0.01	−19.1 ± 1.3
Franz’s Cells	25 °C	117.2 ± 1.4	0.1 ± 0.01	−19.0 ± 1.0
	37 °C	114.3 ± 3.1	0.2 ± 0.01	−16.0 ± 0.5

**Table 5:** Molecules used in the simulation of mixed NLC systems.

Molecules		MD-1 (300 K)	MD-2 (358 K)	MD-3 (358 K)	MD-4 (358 K)
Rhodamine	Rhodamine 123	-	-	-	12
Miglyol^®^ 812	Capric Triglyceride	3	3	-	3
	Caprylic Triglyceride	3	3	-	3
Tween^®^ 80	Polysorbate 80	8	8	8	8
Gelucire^®^ 44/14	Lauric acid PEG	200	200	200	200
	Mono-glyceride Lauric acid	17	17	17	17
	Di-glyceride Lauric acid	17	17	17	17
	Tri-glyceride Lauric acid	17	17	17	17

## Supplementary Materials

The following are available online at https://www.mdpi.com/article/10.3390/pharmaceutics13040531/s1, Table S1: Preliminary formulations leading to the selected NLC (%*w/w*) composition. In blue is the selected formulation composition for the current investigation, Figure S1: Hydrodynamic diameter (nm) and zeta potential of all formulation. The formulations not included in the figure were physically unstable.
