# Supplementary material for: Development of a Nanostructured Lipid Carrier (NLC) by a Low-Energy Method, Comparison of Release Kinetics and Molecular Dynamics Simulation
Source: Pharmaceutics. 2021 Apr 10;13(4):531. doi: 10.3390/pharmaceutics13040531 (PMC8070589; doi:10.3390/pharmaceutics13040531)
Supplement: Supplementary file 1 [file pharmaceutics-13-00531-s001.pdf]

pharmaceutics

Supplementary materials: Development of a Nanostructured Lipid Carrier (NLC) by a low-Energy Method, Comparison of Release Kinetics and Molecular Dynamics Simulation

Andrea C. Ortiz, Osvaldo Yañez, Edison Salas-Huenuleo and Javier O. Morales

Table S1. Preliminary formulations leading to the selected NLC (%p/p) composition. In blue is the selected formulation composition for the current investigation.

Formulation	Gelucire® 44/14	Tocoferol	Stearic acid	Oleic acid	Mygliol® 812	Tween® 80	Span® 20	Water
A1	5	------	------	0.5	------	------	3.5	91
A2	5	------	------	1	------	2	------	92
A3	4	------	------	2.5	------	1.5	1	91
A4	4	------	------	2	------	------	2	92
A5	4	1	------	0.5	------	1.5	------	91
A6	3	2	------	1	------	2	------	92
A7	3	3	------	2	------	2	1	89
A8	4	------	------	------	0.5	2.5	------	93
A9	4	------	------	------	1	2	------	93
A10	4	------	------	------	1.5	2.5	2	90
A11	3	------	1	------	1	2	------	93
A12	4	------	2	------	2	------	2	90

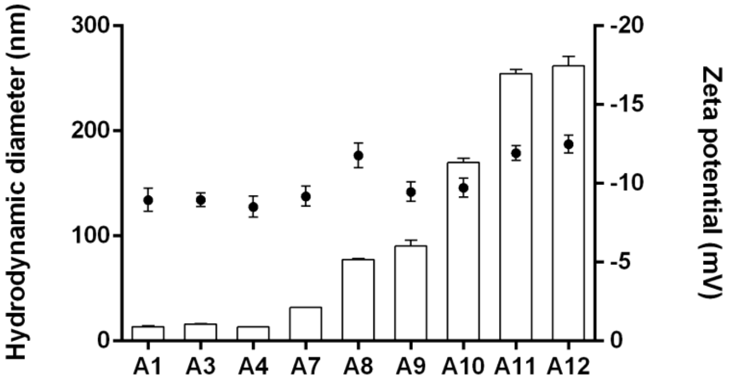

Figure S1. Hydrodynamic diameter (nm) and zeta potential of all formulation. The formulations not included in the figure were physically unstable.
